# Enhanced Oxygen Storage Capacity of Porous CeO_2_ by Rare Earth Doping

**DOI:** 10.3390/molecules28166005

**Published:** 2023-08-10

**Authors:** Yaohui Xu, Liangjuan Gao, Quanhui Hou, Pingkeng Wu, Yunxuan Zhou, Zhao Ding

**Affiliations:** 1 Laboratory for Functional Materials, School of New Energy Materials and Chemistry, Leshan Normal University, Leshan 614000, China; xuyaohui1986@163.com; 2 Leshan West Silicon Materials Photovoltaic and New Energy Industry Technology Research Institute, Leshan 614000, China; 3 College of Materials Science and Engineering, Sichuan University, Chengdu 610065, China; lgao87@scu.edu.cn; 4 School of Automotive Engineering, Yancheng Institute of Technology, Yancheng 224051, China; hqhdyx66@ycit.edu.cn; 5 Department of Chemical Engineering, Illinois Institute of Technology, Chicago, IL 60616, USA; pwu18@hawk.iit.edu; 6 College of Materials Science and Engineering, National Engineering Research Center for Magnesium Alloys, Chongqing University, Chongqing 400044, China

**Keywords:** CeO_2_, porous, rare earth, doping, oxygen storage capacity, solvothermal

## Abstract

CeO_2_ is an important rare earth (RE) oxide and has served as a typical oxygen storage material in practical applications. In the present study, the oxygen storage capacity (OSC) of CeO_2_ was enhanced by doping with other rare earth ions (RE, RE = Yb, Y, Sm and La). A series of Undoped and RE–doped CeO_2_ with different doping levels were synthesized using a solvothermal method following a subsequent calcination process, in which just Ce(NO_3_)_3_∙6H_2_O, RE(NO_3_)_3_∙nH_2_O, ethylene glycol and water were used as raw materials. Surprisingly, the Undoped CeO_2_ was proved to be a porous material with a multilayered special morphology without any additional templates in this work. The lattice parameters of CeO_2_ were refined by the least–squares method with highly pure NaCl as the internal standard for peak position calibrations, and the solubility limits of RE ions into CeO_2_ were determined; the amounts of reducible–reoxidizable Ce^n+^ ions were estimated by fitting the Ce 3d core–levels XPS spectra; the non–stoichiometric oxygen vacancy (*V*_O_) defects of CeO_2_ were analyzed qualitatively and quantitatively by O 1s XPS fitting and Raman scattering; and the OSC was quantified by the amount of H_2_ consumption per gram of CeO_2_ based on hydrogen temperature programmed reduction (H_2_–TPR) measurements. The maximum [OSC] of CeO_2_ appeared at 5 mol.% Yb–, 4 mol.% Y–, 4 mol.% Sm– and 7 mol.% La–doping with the values of 0.444, 0.387, 0.352 and 0.380 mmol H_2_/g by an increase of 93.04, 68.26, 53.04 and 65.22%. Moreover, the dominant factor for promoting the OSC of RE–doped CeO_2_ was analyzed.

## 1. Introduction

Rare earth (RE), known as “Industrial vitamin”, “Industrial monosodium glutamate” and “Mother of new material”, has irreplaceable excellent magnetic, optical, and electrical properties, playing a huge role in improving product performance, increasing product variety and improving production efficiency. Although the amount is small, it can greatly optimize the properties of materials. In view of its large effect and low dosage, RE has become an important national strategic resource in improving product structure, increasing technological content, and promoting industry technological progress, and is broadly utilized in many fields, such as metallurgy, military, petrochemical, glass ceramics, agriculture and new materials, and so on [1,2,3]. Cerium (Ce) is the most abundant RE element in the crust of Earth, which has good redox performance, so that its oxide (cerium oxide, CeO_2_) shows excellent oxygen transport capacity and oxygen storage/release capacity. Moreover, CeO_2_ has the advantages of low toxicity and reusability, so it has attracted great attention in the detection of food biological and chemical substances, catalysis and fuel cell fields [4,5].

Oxygen storage materials are binary or multicomponent composite oxides, in which a CeO_2_ and CeO_2_–based solid solution are the main components. CeO_2_ is a significant N–type semiconductor material with high electrical conductivity, an excellent oxygen storage/release capacity and strong redox activity. Moreover, CeO_2_–based oxygen storage materials are one of the key materials in a three–way catalyst for automobile exhaust purification [6,7], as well as in water–gas–shift [8,9,10], ethanol steam reforming [11,12] and hydrocarbon reforming [13,14,15]. In an oxygen–rich environment, CeO_2_ can capture ambient oxygen into its own lattice, and release these stored oxygen quickly when the oxygen content of the reaction system is reduced. Because of this, CeO_2_–based oxygen storage materials can even determine the performance and service life of a catalyst [16]. Especially in the heterogeneous catalytic reactions, they can regulate the fluctuation of the oxygen content in the reaction system through their own oxygen storage and oxygen release characteristics, which can always maintain the best catalytic effect. This ability of CeO_2_–based composite oxides to store and release oxygen is called its oxygen storage capacity (OSC). The oxygen evolution and absorption equilibrium reaction can be described by Reaction (1) [17,18]:

(1) $$CeO_{2}\overset{Oxygen\;release}{\underset{Oxygen\;storage}{⇄}}Ce_{1-x}^{4+}Ce_{x}^{3+}O_{2-x/2}V_{x/2}+x/4O_{2}$$

where “*V*_O_” represents the oxygen vacancy defects produced via the vacancy compensation mechanism. Interestingly, CeO_2_ can exhibit a large deviation from stoichiometry at low oxygen partial pressure, forming nonstoichiometric oxide CeO_2−*x*_. Even after the loss of oxygen from the lattice and the consequent formation of numerous *V*_O_, CeO_2−*x*_ still retains a fluorite crystal structure [19,20] and captures oxygen by filling the *V*_O_ upon exposure to oxygen, accompanied by the recovery of CeO_2_ [21]. Moreover, the doping of other metallic elements into the CeO_2_ lattice could control their structure and physical properties [22,23,24], such as rare–earth elements [25,26,27], transition elements [28,29,30] and alkaline earth elements [31,32,33]. In spite of the successful synthesis of CeO_2_–based composite oxides, most of the previous reports have focused on the investigation of catalytic performances [34,35], transport properties [36,37] and the origin of room–temperature ferromagnetism [38,39], the theoretical data about OSC were usually quite scattered, and only a few fundamental studies on the OSC of doped CeO_2_ have been reported. For example, Singh [40] et al. synthesized a series of Ce_1−*x*_M*_x_*O_2−σ_ (M = Zr, Ti, Pr, Y and Fe) nanocrystallites using the hydrothermal method using melamine and diethylenetriamine as complexing agents; up to 50% Zr and Y, 40% Ti, 25% Pr and 15% Fe were substituted for Ce^4+^ in CeO_2_, and Ce_0.85_Fe_0.15_O_1.85_ showed a higher OSC and higher CO conversion at a lower temperature than Ce_1−x_Zr_x_O_2_. Ansari et al. [41] reported the redox properties of Fe–doped CeO_2_ nanoparticles obtained by a polyol–assisted co–precipitation process, and the 10 mol.% Fe doped CeO_2_ nanoparticles exhibited excellent reduction performance. Si et al. [42] prepared Ce_1−*x*_Zr*_x_*O_2_ (*x* = 0~0.8) powders via a mild urea hydrolysis based on the hydrothermal method, and validated a linear relationship between the lattice strain and the OSC value of CeO_2_–ZrO_2_ solid solutions. Therefore, the microstructure and OSC of doped CeO_2_ have to be understood at a fundamental level through a series of dopants to design advanced materials.

For that, four rare earth elements (RE = Yb, Y, Sm and La) were selected as dopants to improve the OSC of CeO_2_ based on the similarity–intermiscibility theory. In order to avoid the influence of other ions on the doping effect, we only used Ce(NO_3_)_3_∙6H_2_O, RE(NO_3_)_3_∙nH_2_O, ethylene glycol and water as raw materials. Moreover, all experimental conditions and the purity of raw materials were the same, so, the comparison of structure and properties of RE–doped CeO_2_ was reliable and effective. Based on this, the influence of the dopant elements and their amounts on the non–stoichiometric *V*_O_ and OSC were investigated and discussed. Surprisingly, the undoped CeO_2_ was proved to be a porous material with a multilayered morphology without any additional templates, and the effect of RE–doping on morphology of CeO_2_ also was investigated.

## 2. Experimental Procedure

### 2.1. Starting Materials

Ce(NO_3_)_3_∙6H_2_O (99.95%), Yb(NO_3_)_3_∙5H_2_O (99.9%), Y(NO_3_)_3_∙6H_2_O (99.9%), Sm(NO_3_)_3_∙6H_2_O (99.9%) and La(NO_3_)_3_∙6H_2_O (99.9%) were supplied by Aladdin Co., Ltd. (Shanghai, China). Ethylene glycol (99.5%) and ethanol (99.7%) were obtained from Chengdu Kelong Chemical Co., Ltd. (Chengdu, China). Distilled water was used in all experiments.

### 2.2. Synthesis of Undoped and RE–Doped CeO_2_

Firstly, the desired amounts of Ce(NO_3_)_3_∙6H_2_O and RE(NO_3_)_3_∙nH_2_O (RE = Yb, Y, Sm and La) with different RE/(RE + Ce) (mol.%) were dissolved in a mixed solution of 25 mL ethylene glycol and 5 mL distilled water, the total amount of Ce^3+^ and RE^3+^ ions was 4.0 mmol. Then, the mixed solution was decanted into a 50 mL Teflon–lined stainless steel autoclave and sealed. Subsequently, the solvothermal process lasted for 24 h at 200 °C. After the reaction, the resulting precipitates were collected by centrifugation, and washed thrice alternately with distilled water and ethanol. At this point, the precursors synthesized by the hydrothermal process were obtained after drying in air at 80 °C for 12 h. Finally, a series of RE–doped CeO_2_ powders were obtained by following calcination in air at 500 °C for 2 h. For comparison, the Undoped CeO_2_ was synthesized using the same procedure, albeit in the absence of dopants RE(NO_3_)_3_∙nH_2_O.

### 2.3. Characterization

The actual doping amounts of RE elements in CeO_2_ were determined using an inductively coupled plasma–atomic emission spectrometer (ICP–AES, SPECTRO ARCOS EOP, Kleve, Germany). The crystallographic phases of samples were characterized by X-ray diffraction (XRD, Rigaku D/MAX 2200 PC, Rigaku, Japan) analysis using graphite monochromatized Cu Karadiation with 40 kV tube voltages and a 40 mA current. The morphologies of CeO_2_ were observed by field–emission scanning electron microscopy (SEM, JEOL–7500F, Tokyo, Japan). The surface composition and binding energy of CeO_2_ were determined by X-ray photoelectron spectroscopy (XPS, ESCALAB 250, Thermo Scientific, Waltham, MA, USA). The natures of surface *V*_O_ defects were identified using Raman spectroscopy (LabRAM Aramis, Horiba Jobin Yvon, Paris, France) with a He–Cd laser of 325 nm. N_2_ adsorption–desorption isotherms were measured on a QuadraSorb SI (Quantachrome, Boynton Beach, FL, USA), and the specific surface areas were determined using the Brunauer–Emmett–Teller method.

### 2.4. Evaluation of OSC

For the Undoped and RE–doped CeO_2_ samples synthesized using the hydrothermal process at 200 °C for 24 h, followed by calcination in air at 500 °C for 2 h, the hydrogen temperature programmed reduction (H_2_–TPR) measurements were employed to evaluate their OSC, which was carried out on a TP–5080 instrument with a thermal conductivity detector of gas chromatography. Typically, 50 mg CeO_2_ powder was pre–treated in a 5% O_2_/N_2_ stream at 500 °C for 1 h. After cooling down, the sample was purged with N_2_ to remove the excess O_2_. Then, a flow of 5% H_2_/N_2_ was introduced into the reactor with a flow rate of 30 mL/min, and the temperature was raised to ~650 °C with a heating rate of 10 °C/min.

## 3. Results and Discussion

**Figure 1:**
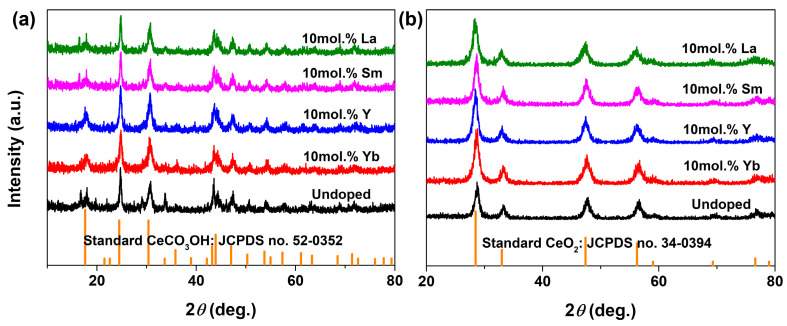
XRD patterns of the Undoped and 10 mol.% RE–doped samples synthesized using a solvothermal process at 200 °C for 24 h (**a**) before and (**b**) after calcination in air at 500 °C for 2 h.

XRD analyses were employed to identify the phase composition and crystallographic structure of the as–obtained precursors and samples. Figure 1a showed the XRD patterns of the precursors synthesized using the solvothermal process at 200 °C for 24 h before calcination. For the precursor synthesized without RE, its major phase component was CeCO_3_OH (JCPDS no. 52–0352), and similar profiles were observed for these precursors synthesized with the introduction of 10 mol.% RE in the solvothermal process. Figure 1b showed the XRD patterns of Undoped and 10 mol.% RE–doped samples synthesized at 200 °C for 24 h after calcination in air at 500 °C for 2 h. All identified peaks had a good match with the standard CeO_2_ pattern (cubic fluorite structure, JCPDS no. 34–0349), and the intensities of the corresponding diffraction peaks were comparable. Moreover, no impurity phases were detected, such as Yb_3_O_4_, Y_3_O_4_, Sm_3_O_4_ and La_3_O_4_. The absence of RE impurity phases could be explained as follows. The RE impurity phases in the sample might exist as highly dispersed amorphous species. Another possibility was that the RE impurity ions partially substituted the host Ce ions to form a solid solution. Compared with Undoped CeO_2_, the relative diffraction intensities of 10 mol.% RE–doped CeO_2_ showed no clear differences, suggesting that there was no preferential orientation or preferential crystal growth upon the incorporation of RE. In addition, compared with Undoped CeO_2_, a recognizable peak shift towards lower diffraction angles for 10 mol.% RE–doped CeO_2_ was observed. These findings indicate that the larger RE impurity ions partially substituted the host Ce ions to form the RE–based solid solution based on Bragg’s equation, and the cubic fluorite crystal structure of CeO_2_ was maintained.

**Figure 2:**
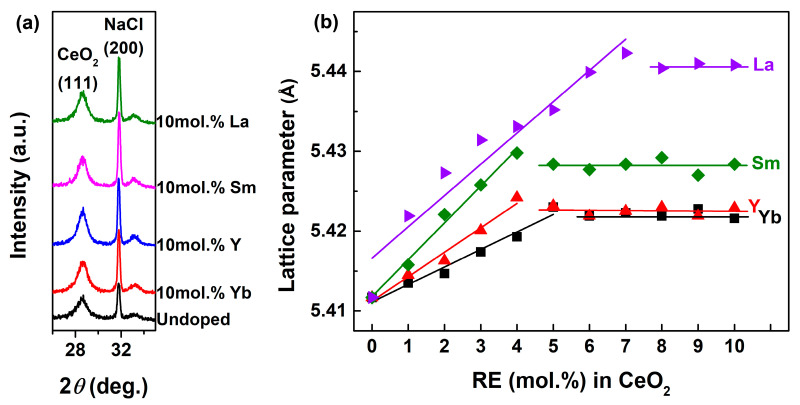
(**a**) XRD patterns of Undoped and 10 mol.% RE–doped CeO_2_ with the internal standard of NaCl, (**b**) lattice parameter and fitting curves of RE–doped CeO_2_ (RE = Yb, Y, Sm and La, [RE] ≤ 10 mol.%). The RE (mol.%) in CeO_2_ = 0 represented the Undoped CeO_2_ sample.

When the impurity ions were introduced into the lattice of the matrix, its lattice parameter (*a*) would change. So, the change in the *a* value could be used to determine the solubility limit of these dopants in the matrix. In this work, the *a* value of CeO_2_ was refined by the least–squares method, in which the highly pure NaCl (≥99.999%) was selected as an internal standard to calibrate the peak position of CeO_2_. Figure 2a showed the XRD patterns of Undoped CeO_2_ and 10 mol.% RE–doped CeO_2_ with the internal standard of NaCl. It could be found that the diffraction intensities of (111) peak from CeO_2_ and (200) peak from NaCl were comparable, suggesting the feasibility of this internal standard method. Moreover, the *a* values of Undoped and 1~10 mol.% RE–doped CeO_2_ were calculated, and the calculated *a* as a function of RE contents in CeO_2_ were summarized in Figure 2b. From Figure 2b, the *a* values of all RE–doped CeO_2_ were greater than that of the Undoped one (5.4117 Å). Under the same doping concentration, the variation trend of *a* values was as follows: *a*_Yb_ < *a*_Y_ < *a*_Sm_ < *a*_La_, which was consistent with the sequence of their ionic radii for CN8: *R*_Ce_ (0.97 Å) < *R*_Yb_ (0.98 Å) < *R*_Y_ (1.02 Å) < *R*_Sm_ (1.08 Å) < *R*_La_ (1.16 Å) according to Shannon’s compilation [43]. The increased *a* values after the introduction of RE indicated that the partial host Ce^4+^ (0.97 Å) ions substituted by the larger RE ions and the local lattice expansion of CeO_2_ crystal occurred as a result. Moreover, the *a* values linearly increased with increasing RE contents, reached a maximum at 5, 4, 4 and 7 mol.% for Yb, Y, Sm and La, before decreasing and maintaining a certain level for higher RE contents. This would indicated that the solubility limits of Yb, Y, Sm and La ions in CeO_2_ were 5, 4, 4 and 7 mol.%.

**Figure 3:**
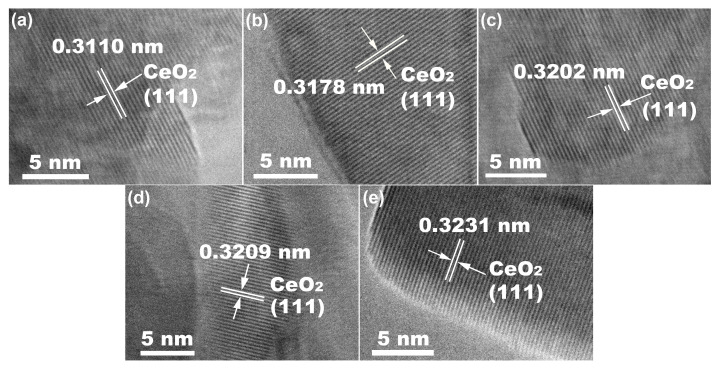
HR–TEM images of (**a**) Undoped CeO_2_ and 10 mol.% (**b**) Yb, (**c**) Y, (**d**) Sm and (**e**) La–doped CeO_2_ synthesized using the hydrothermal process at 200 °C for 24 h and followed by calcination in air at 500 °C for 2 h.

In order to further confirm the incorporation of RE ions and their effect on the CeO_2_ lattice, high–resolution electron microscopy (HR–TEM) was performed and the corresponding HR–TEM images of Undoped and 10 mol.% RE–doped CeO_2_ were synthesized using the hydrothermal process at 200 °C for 24 h, followed by calcination in air at 500 °C for 2 h, as shown in Figure 3. From the HR–TEM image of Undoped CeO_2_ in Figure 3a, the interplanar spacing was measured with a value of 0.3110 nm, which fitted well with the (111) plane of cubic CeO_2_, proving the generation of the CeO_2_ phase. After the incorporation of 10 mol.% RE (RE = Yb, Y, Sm and La), the interplanar spacings of CeO_2_ in Figure 3b–e had increased to 0.3178, 0.3202, 0.3209 and 0.3231 nm, respectively. Combined with XRD analysis results in Figure 2, both the local lattice expansion and the increased interplanar spacing indicated that these large RE (*R*_Yb_ = 0.98 Å; *R*_Y_ = 1.02 Å; *R*_Sm_ = 1.08 Å; *R*_La_ = 1.16 Å) impurity ions partially substituted the host Ce ions (*R*_Ce_ = 0.97 Å), and a solid solution was formed. Importantly, the size of the RE impurity ions was consistent with the trends of interplanar spacing. In other words, the larger the size of the doped RE ion, the greater the interplanar spacing of the as–obtained RE–doped CeO_2_. In addition, the practical RE contents in CeO_2_ were measured by ICP–AES, and the results are shown in Table 1. As observed in Table 1, it could be found that the practical RE contents in CeO_2_ were close to the corresponding nominal doped one.

**Figure 4:**
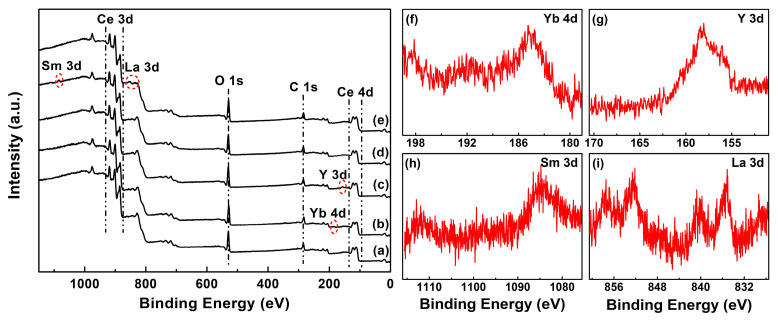
Full–range XPS spectra of (**a**) Undoped, 4 mol.% (**b**) Yb, (**c**) Y, (**d**) Sm and (**e**) La–doped CeO_2_ synthesized using the hydrothermal process at 200 °C for 24 h and followed by calcination in air at 500 °C for 2 h; corresponding XPS regions of (**f**) Yb 4d, (**g**) Y 3d, (**h**) Sm 3d and (**i**) La 3d.

XPS analysis was employed to probe the surface chemical composition and various oxidation states before and after RE–doping. Figure 4a–e shows the wide–scan XPS spectra of Undoped and 4 mol.% RE–doped CeO_2_ synthesized using the hydrothermal process at 200 °C for 24 h and followed by calcination in air at 500 °C for 2 h, respectively. As observed, all wide–scan XPS spectra showed the clear CeO_2_ features by the signals of Ce 3d, Ce 4d and O 1s, in good agreement with those XPS patterns of Gd– [44], Y– [45] and Dy– [46] doped CeO_2_. It is worth noting that the obvious C 1s peaks located at ~284.8 eV were derived from adventitious carbon to calibrate the tested samples. Moreover, the faint RE 3d or RE 4d signals can be seen in the red dotted box in Figure 4b–e, and the corresponding Yb 4d, Y 3d, Sm 3d and La 3d XPS regions were recorded, as shown in Figure 4f–i, respectively. The characteristic peaks in Figure 4f–i implied that the Yb, Y, Sm and La elements were in +3 states. It indicated that the Yb, Y, Sm and La elements had been successfully incorporated into the CeO_2_ lattice with positive trivalent states (RE^3+^).

**Figure 5:**
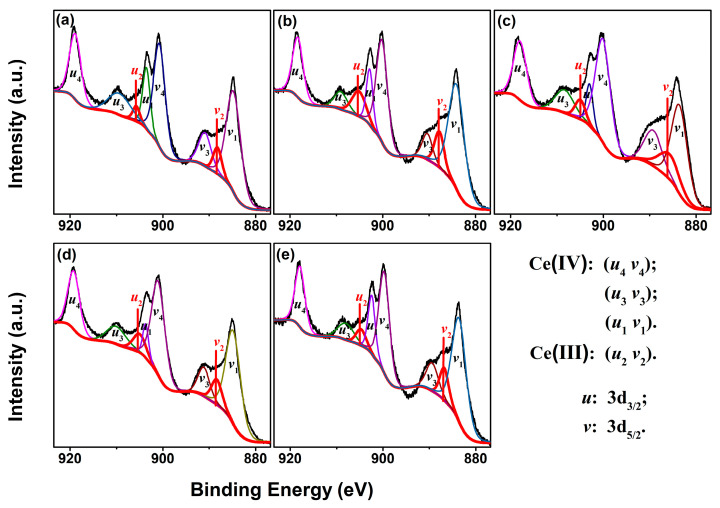
Ce 3d core–levels XPS spectra of (**a**) Undoped, 4 mol.% (**b**) Yb, (**c**) Y, (**d**) Sm and (**e**) La–doped CeO_2_ synthesized using the hydrothermal process at 200 °C for 24 h and followed by calcination in air at 500 °C for 2 h.

In order to understand the effect of RE–doping on Ce ions in the CeO_2_ crystal, the Ce 3d XPS regions of Undoped and 4 mol.% RE–doped CeO_2_ were recorded and fitted, as shown in Figure 5a–e. The Ce 3d XPS core–levels of all CeO_2_ samples were fitted into eight peaks, corresponding to four pairs of spin–orbit doublets (*u*_1–4_ and *v*_1–4_) of Ce ions, in which the *u*_i_ and *v*_i_ bands corresponded to the contributions of Ce 3d_3/2_ and Ce 3d_5/2_. Moreover, the bands of *u*_4_, *u*_3_ and *u*_1_ (and those for *v*_4_, *v*_3_, *v*_1_) were attributed to the Ce^4+^ state, while *u*_2_ and *v*_2_ were due to the Ce^3+^ state [47]. Meanwhile, the relative concentration of Ce^3+^ ions in CeO_2_, labeled as [Ce^3+^]_XPS_, could be calculated by the ratio of integrated peak areas of the peak related to the Ce^3+^ species (*u*_2_ and *v*_2_ peaks) to that of all peaks (*u*_1–4_ and *v*_1–4_ peaks) in Figure 5, and the results were summarized in Table 2. As observed, the [Ce^3+^]_XPS_ values of 4 mol.% Yb, Y, Sm and La–doped CeO_2_ were 13.78, 12.60, 10.94 and 9.78%, respectively, higher than that of Undoped CeO_2_ (6.54%), which indicates that Undoped CeO_2_ itself contained a certain number of Ce^3+^ ions, and RE–doping could promote the formation of Ce^3+^ species. In other words, the amount of reducible–reoxidizable Ce^n+^ (namely, Ce^3+^ ⇔ Ce^4+^) ions increased with the introduction of RE ions into CeO_2_ lattice, indicating that RE–doping was conducive to improving the redox capacity of CeO_2_. 

**Figure 6:**
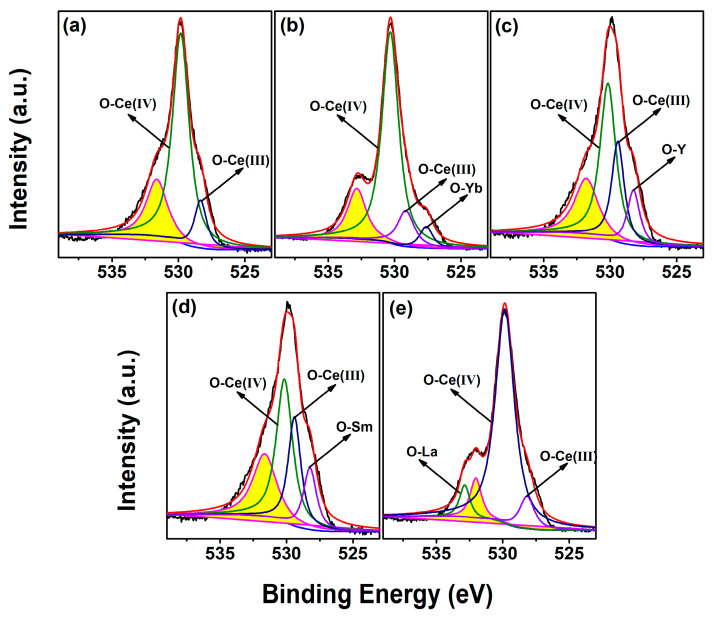
O 1s core–level XPS spectra of (**a**) Undoped, 4 mol.% (**b**) Yb, (**c**) Y, (**d**) Sm and (**e**) La–doped CeO_2_ synthesized using the hydrothermal process at 200 °C for 24 h and followed by calcination in air at 500 °C for 2 h.

To investigate the chemical states of O in CeO_2_, the O 1s core–level XPS spectra of Undoped and 4 mol.% RE–doped CeO_2_ were recorded and fitted, as shown in Figure 6a–e. For Undoped CeO_2_ in Figure 6a, its O 1s XPS spectrum could be curve–fitted into three peaks, indicating the presence of three kinds of oxygen species in CeO_2_. The peaks with a binding energy of ~529.8 and ~528.4 eV could be assigned to lattice oxygen of O–Ce(IV) species and O–Ce(III) species, respectively, whereas that of ~531.6 eV (yellow region peak) could be assigned to the chemisorption of oxygen or/and weakly bonded oxygen species related to *V*_O_ defects. For the O 1s spectra of RE–doped CeO_2_ in Figure 6b–e, besides the above three peaks, a new curve fitting could be observed, which might be attributed to the corresponding O–RE species, namely, the O–Yb species at ~527.6 eV, O–Y species at ~528.2 eV, O–Sm species at ~528.2 eV and O–La species at ~532.9 eV. Furthermore, the relative *V*_O_ content could be estimated by the ratio of the integrated area of the peak related to the *V*_O_ defect (yellow region peak in Figure 6a–e) to that of all peaks, labeled as [*V*_O_]_XPS_, and the results were summarized in Table 2. As observed in Table 2, the calculated [*V*_O_]_XPS_ values of 4 mol.% Yb, Y, Sm and La–doped CeO_2_ were 30.00, 26.82, 26.81 and 17.28%, respectively, higher than that of the Undoped one (13.42%). This result indicated that RE–doping was beneficial for the *V*_O_ creation in CeO_2_.

From the results of XPS analyses in Figure 4, Figure 5 and Figure 6 and Table 2, it could be concluded that RE elements were successfully incorporated into the CeO_2_ lattice with positive trivalent states, and RE–doping could increase the amount of redox Ce^n+^ (Ce^3+^/Ce^4+^) of CeO_2_, as well as the *V*_O_ defects.

**Figure 7:**
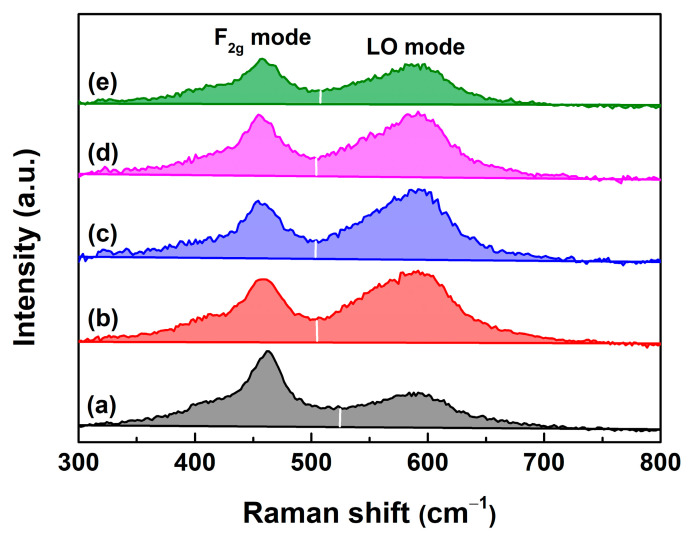
Raman spectra of (**a**) Undoped, 4 mol.% (**b**) Yb, (**c**) Y, (**d**) Sm and (**e**) La–doped CeO_2_ synthesized using the hydrothermal process at 200 °C for 24 h and followed by calcination in air at 500 °C for 2 h.

Due to its sensitivity to the *V*_O_ defect, Raman scattering was employed to investigate the structure of Undoped and RE–doped CeO_2_ synthesized using the hydrothermal process at 200 °C for 24 h and followed by calcination in air at 500 °C for 2 h [48,49]. For the Undoped CeO_2_ in Figure 7a, the peak at ~458 cm^−1^ was attributed to the triply degenerate F_2g_ mode from the symmetric O–Ce–O stretching mode [50], while the weak peak at ~592 cm^−1^ was assigned to the optical LO mode related to *V*_O_ defects [51,52,53]. Upon the incorporation of RE^3+^ ions into the CeO_2_ lattice, the band intensity of the F_2g_ mode decreased, while that of the LO mode related to the *V*_O_ defect increased (Figure 7b–e). It indicated that Undoped CeO_2_ itself had a certain number of *V*_O_ defects and RE–doping could favor the presence of substoichiometric CeO_2−x_ underscoring an increase in *V*_O_ defects, as consistent with the analysis results of O 1s core–level XPS spectra in Figure 6.

**Figure 8:**
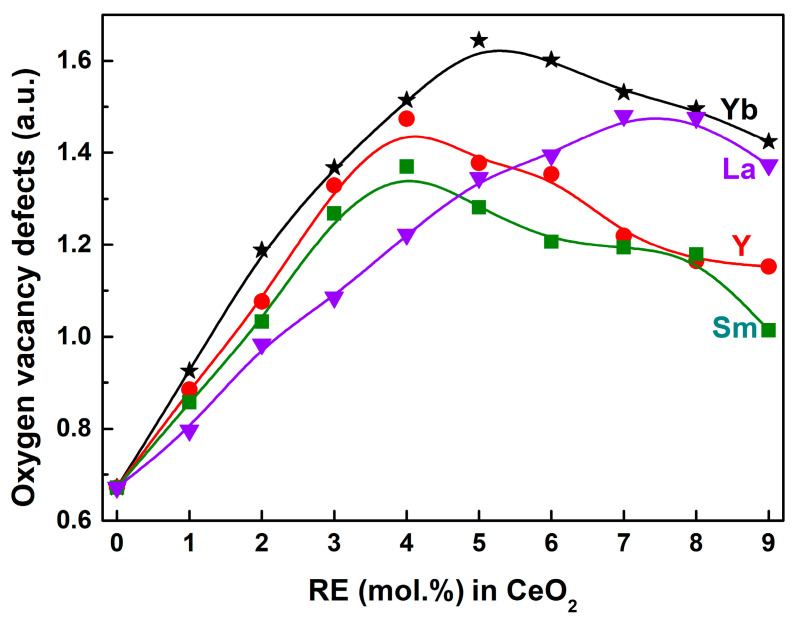
Relative *V*_O_ concentrations of 0~9 mol.% RE–doped CeO_2_ calculated using integral area ratio from Raman spectra (RE = Yb, Y, Sm and La). The RE (mol.%) in CeO_2_ = 0 represented the Undoped CeO_2_ sample.

The band at ~590 cm^−1^ in Raman spectra was known to be associated with the *V*_O_ defect and has been widely observed in substoichiometric CeO_2−x_ [54]. From Figure 7a, the band intensity of both the F_2g_ and LO modes obviously changed upon the incorporation of RE^3+^ ions into the CeO_2_ lattice, which was attributed to the increased lattice distortion caused by RE–doping and hence interfered with the vibrations of CeO_2−x_. It made the quantitative analysis of *V*_O_ defects difficult. For this, an alternative approach to quantitatively estimate the relative contents of *V*_O_ defects was adopted by the ratio of the integrated area of the LO mode to that of the F_2g_ mode from the Raman spectra. Figure 8 showed the calculated relative *V*_O_ concentrations of Undoped and 1~9 mol.% RE–doped CeO_2_ synthesized by the hydrothermal process at 200 °C for 24 h and followed by calcination in air at 500 °C for 2 h. As observed, there existed a certain amount of *V*_O_ defects in Undoped CeO_2_, and the calculated value was 0.67, consistent with the analysis results of the O 1s core–level XPS spectra in Figure 6. These intrinsic *V*_O_ defects might have evolved from the redox cycle of Ce^n+^ in CeO_2_ (Ce^3+^ ⇔ Ce^4+^). The relative *V*_O_ concentrations increased almost linearly with increasing RE contents, and reached maximum when the RE contents were 5, 4, 4 and 7 mol.% for Yb, Y, Sm and La–doped CeO_2_, and gradually decreased above this doping level. Before this turning point, the variation trend of relative *V*_O_ concentration under the same doping concentration was as follows: Yb > Y > Sm > La, which was consistent with their electronegativity: *χ*_Yb_ (1.26) > *χ*_Y_ (1.22) > *χ*_Sm_ (1.17) > *χ*_Ce_ (1.12) > *χ*_La_ (1.11). After the RE^3+^ ions substituted the host Ce ions into the CeO_2_ lattice, the bigger its electronegativity, the stronger its ability to attract the surrounding electrons to itself, and the surrounding O^2−^ anions lost electrons more easily, thus resulting in extrinsic *V*_O_ defects.

**Figure 9:**
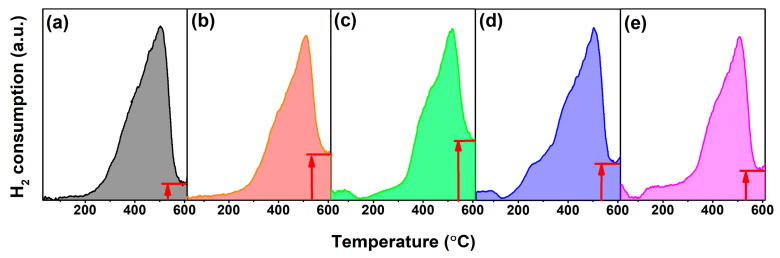
H_2_–TPR profiles of (**a**) Undoped, 4 mol.% (**b**) Yb, (**c**) Y, (**d**) Sm and (**e**) La–doped CeO_2_ synthesized using the hydrothermal process at 200 °C for 24 h and followed by calcination in air at 500 °C for 2 h. (30 mL/min 5%–H_2_/N_2_ flow; Heating rate 10 °C/min).

H_2_–TPR measurements were employed to evaluate the OSC of CeO_2_. Figure 9a–e illustrated the H_2_–TPR profiles of Undoped and 4 mol.% RE–doped CeO_2_ (RE = Yb, Y, Sm and La) synthesized using the hydrothermal process at 200 °C for 24 h and followed by calcination in air at 500 °C for 2 h. For all CeO_2_ samples in Figure 9, one can clearly find a distinct H_2_ reduction band from 30 to 610 °C, with the strongest H_2_ reduction peak at ~510 °C; the maximum H_2_ consumption occurred at 510 °C and then decreased until ~600 °C, and after that it tended to rise. The reduction band from 30 °C to ~600 °C could be attributed to the reduction in surface/subsurface lattice oxygen, which was consistent with these reported results [55,56]. Before 200 °C, the RE–doped CeO_2_ in Figure 9b–e exhibited more H_2_ consumption than that of the Undoped CeO_2_; especially for 4 mol.% Y, Sm and La–doped CeO_2_, a minima at 170 °C occurred. This indicated that the specific surface area of CeO_2_ played a dominant role in its OSC at low temperatures. To prove this conjecture, we tested the specific surface areas of 4 mol.% Yb, Y, Sm and La–doped CeO_2_, and the results were summarized in Table 2. The specific surface areas of 4 mol.% Y, Sm and La–doped CeO_2_ were 98.1, 112.6 and 104.6 m^2^/g, respectively, higher than that of Undoped CeO_2_ (96.0 m^2^/g); however, these decreased after 4 mol.% Yb–doping (89.7 m^2^/g). Moreover, compared to Undoped CeO_2_ in Figure 9a, there appeared to be a visible shoulder from ~350 °C in the H_2_–TPR profiles of RE–doped CeO_2_ in Figure 9b–e, and the reduction bands of RE–doped CeO_2_ at ~600 °C were far higher than the baseline. These phenomena suggested that RE–doping optimized the surface states of CeO_2_, thereby enhancing its OSC.

**Figure 10:**
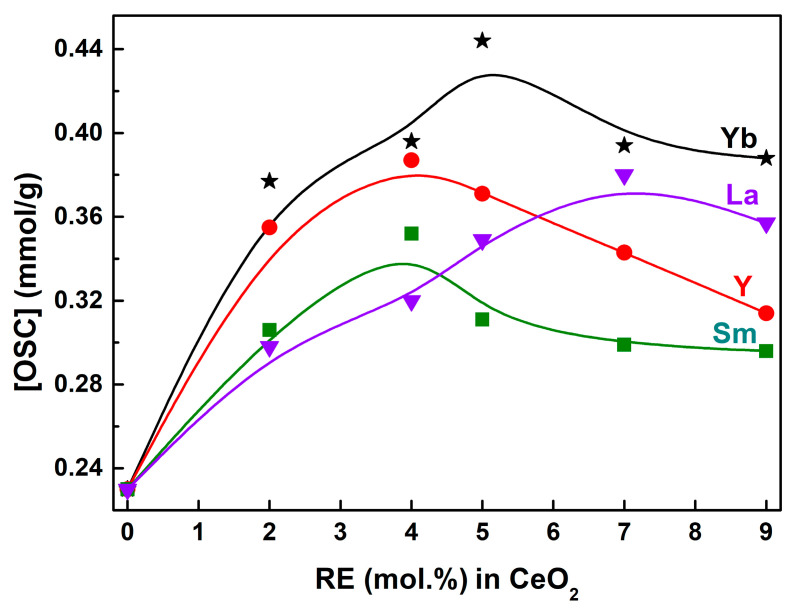
Relative [OSC] values of 0~9 mol.% RE–doped CeO_2_ calculated by measuring the corresponding peak areas of H_2_–TPR profiles (RE = Yb, Y, Sm and La). Note: [OSC] was the value of quantified OSC using the amount of H_2_ consumption per gram of CeO_2_ powders (mmol H_2_/g CeO_2_) by measuring the corresponding peak areas of H_2_–TPR profiles from 30 °C to ~600 °C.

OSC was the fundamental performance of CeO_2_ and CeO_2_–based oxygen storage materials; so, the quantification of OSC was the key to evaluate their oxygen storage/release property. For that, the OSC was quantified using the amount of H_2_ consumption per gram of CeO_2_ powders by measuring the corresponding peak areas of H_2_–TPR profiles in this work. The quantified OSC (labeled as [OSC], mmol H_2_/g CeO_2_) from 30 °C to ~600 °C, which was the value of H_2_ consumption per gram of CeO_2_ powders, is shown in Figure 10. The [OSC] of Undoped CeO_2_ was 0.23 mmol H_2_/g, indicating that pure CeO_2_ itself possessed a certain OSC, which was attributed to the unique structure of its intrinsic *V*_O_ defect or the redox cycle of Ce^3+^ ⇔ Ce^4+^, supported by the XPS analyses in Figure 5 and Figure 6 and Raman analyses in Figure 7 and Figure 8. For Yb–doped CeO_2_, the [OSC] value reached a maximum with a doping level of 5 mol.% and decreased at a higher Yb content. Interestingly, Y–, Sm– and La–doped CeO_2_ also showed similar trends, reaching the maximum H_2_ consumptions with doping contents of 4, 4 and 7 mol.%, respectively. The [OSC] values of 5 mol.% Yb–, 4 mol.% Y–, 4 mol.% Sm– and 7 mol.% La–doped CeO_2_ were 0.444, 0.387, 0.352 and 0.380 mmol H_2_/g, with an increase of 93.04, 68.26, 53.04 and 65.22% compared with that of the Undoped one (0.230 mmol H_2_/g). These findings indicate that RE–doping could effectively improve the OSC of CeO_2_, combined with the H_2_–TPR curves. This enhanced OSC of RE–doped CeO_2_ could be explained as follows. When RE^3+^ ions were doped into the CeO_2_ lattice to substitute host Ce^4+^ ions, more *V*_O_ defects would be generated to keep the electric neutrality of the fluorite structure, and a substoichiometric solid solution Ce_1–_*_x_*RE*_x_*O_2−σ_ (RE = Yb, Y, Sm and La) was formed based on RE–doping. During the H_2_ reduction of H_2_–TPR, H_2_ reacted with a chemisorbed oxygen from the CeO_2_ surface, which was fixed by intrinsic and extrinsic *V*_O_ defects on the CeO_2_ surface. As the surface chemisorbed oxygen was gradually consumed, the intrinsic and extrinsic *V*_O_ defects were exposed, and the bulk lattice oxygen began to move to the CeO_2_ surface for replenishment by the *V*_O_ defects. The oxygen in the bulk RE–doped CeO_2_ diffused more easily to the surface to fill the *V*_O_ defects than that in the Undoped CeO_2_ due to the activation effect of RE^3+^ dopants which induced oxygen mobility [57].

**Figure 11:**
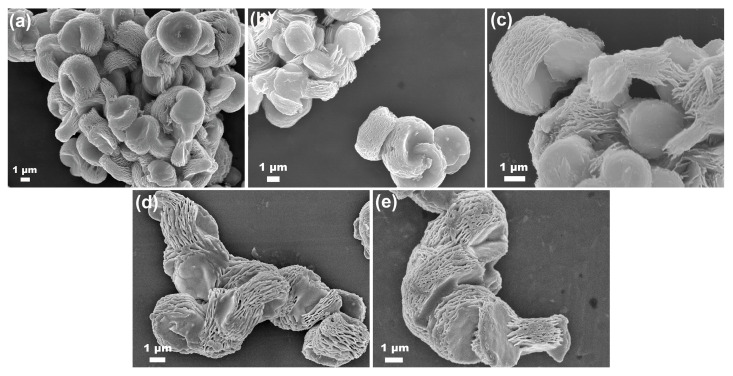
SEM images of (**a**) Undoped, 10 mol.% (**b**) Yb, (**c**) Y, (**d**) Sm and (**e**) La–doped CeO_2_ synthesized using the hydrothermal process at 200 °C for 24 h and followed by calcination in air at 500 °C for 2 h.

In order to investigate the effect of RE–doping on the morphology of CeO_2_, SEM was employed. Figure 11a–e showed the SEM images of Undoped and 10 mol.% Yb, Y, Sm and La–doped CeO_2_ particles synthesized using the hydrothermal process at 200 °C for 24 h and followed by calcination in air at 500 °C for 2 h, respectively. From Figure 11a, it could be seen that the morphology of the Undoped CeO_2_ particle was a multilayered structure consisting of flakes, and these flakes intertwined to form an open porous structure. After the incorporation of 10 mol.% RE (RE = Yb, Y, Sm and La) into CeO_2_, the multilayered morphology was still maintained, as seen in Figure 11b–e. This finding indicates that the low concentration of RE–doping had little effect on the morphology of CeO_2_. Generally, CeO_2_ with a porous structure or special morphology was usually synthesized by a template–based method, in which either surfactants as soft templates or other porous inorganic material as hard templates were used. Surprisingly, the porous CeO_2_ with a multilayered morphology was obtained without any additional templates in this work. The abundant porous structure and highly specific surface area would undoubtedly enhance the OSC of CeO_2_. Further analysis of the porous structures was conducted using an N_2_ adsorption–desorption isotherm, as discussed later.

**Figure 12:**
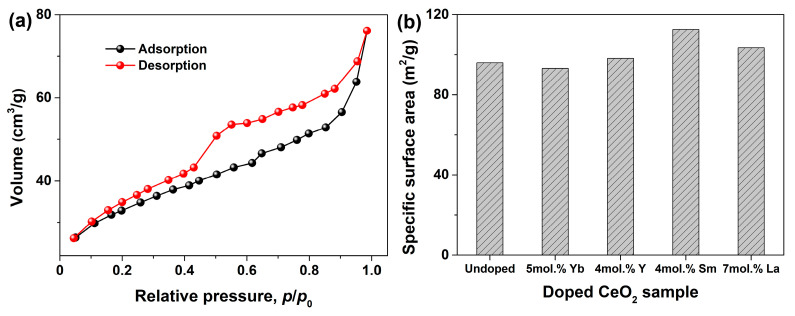
(**a**) N_2_ adsorption–desorption isotherm of Undoped CeO_2_, (**b**) specific surface areas of Undoped, 5 mol.% Yb–doped, 4 mol.% Y–doped, 5 mol.% Sm–doped, 7 mol.% La–doped CeO_2_ synthesized using the hydrothermal process at 200 °C for 24 h and followed by calcination in air at 500 °C for 2 h. Note: Specific surface areas were determined based on N_2_ sorption experiment using the Brunauer–Emmett–Teller method.

In order to further demonstrate the porous structure of CeO_2_, an N_2_ adsorption–desorption experiment was performed, and the N_2_ adsorption–desorption isotherm of Undoped CeO_2_ is shown in Figure 12a. As observed in Figure 12a, the isotherm was similar to the Langmuir IV(a) type according to the IUPAC classification, and an obvious hysteresis loop was observed in the relative pressure range of 0.4~1.0, attributable to the type H3. It suggests that Undoped CeO_2_ was a mesoporous material with a disordered mesoporous structures [58], and the isotherm was consistent with that of other reported porous CeO_2_ [59,60,61]. Moreover, the specific surface areas of Undoped CeO_2_ and RE–doped CeO_2_ with solubility limits were estimated based on the N_2_ adsorption–desorption experiment using a Brunauer–Emmett–Teller method, and the results are shown in Figure 12b as a histogram. Combined with the specific surface areas of 4 mol.% RE–doped CeO_2_ in Table 2, it can be found that RE–doping had a certain influence on the specific surface area of CeO_2_. However, the specific surface area was not the dominant factor for promoting the OSC of RE–doped CeO_2_. Among the CeO_2_ samples with 4 mol.% RE–doping, 4 mol.% Sm–doped CeO_2_ displayed the minimum [OSC] value of 0.352 mmol H_2_/g in Figure 10; however, it possessed the maximum specific surface area of 112.6 m^2^/g in Table 2. Among RE–doped CeO_2_ with saturation doping concentration, 5 mol.% Yb–doped CeO_2_ exhibited the minimum specific surface area of 93.1 m^2^/g in Figure 12b; however, it possessed the maximum [OSC] value of 0.444 mmol H_2_/g. Alternatively, the morphology was also not a major factor influencing the OSC of RE–doped CeO_2_, which is supported by the similar multilayered morphology in Figure 11a–e. Combined with the analyses of morphology and specific surface area of Undoped and RE–doped CeO_2_, one conclusion could be drawn that the enhanced OSC might be attributed to the incorporation of positive trivalent RE^3+^ ions into the CeO_2_ lattice, and partially substituted the host Ce^4+^ ions, promoting the formation of more *V*_O_ defects and the oxidation/reduction cycle of Ce^3+^ ⇔ Ce^4+^. This result could be supported by the lattice parameter analysis in Figure 2, the O 1s XPS analysis in Figure 6 and the Raman spectra analysis in Figure 7.

## 4. Conclusions

In summary, a series of RE–substituted CeO_2_ was synthesized just using Ce(NO_3_)_3_∙6H_2_O, RE(NO_3_)_3_∙nH_2_O (RE = Yb, Y, Sm and La), ethylene glycol and water as raw materials. The Undoped CeO_2_ was proved to be a mesoporous material with a multilayered morphology; both its multilayered morphology and cubic fluorite structure could be maintained even after 10 mol.% RE introduction. The RE elements were successfully incorporated into the CeO_2_ lattice with positive trivalent states. RE–doping was beneficial for the oxidation/reduction cycle of Ce^3+^ ⇔ Ce^4+^, as well as the creation of extrinsic *V*_O_ defects. The solubility limits of Yb, Y, Sm and La ions in CeO_2_ were determined as 5, 4, 4 and 7 mol.%. After the incorporation of larger RE^3+^, the lattice expansion of the CeO_2_ crystal occurred, and more *V*_O_ defects appeared, which could induce the oxygen mobility from bulk to surface, and promote its OSC. The [OSC] values were 0.444, 0.387, 0.352 and 0.380 mmol/g, much higher than that of the Undoped one (0.230 mmol/g), with an increase of 93.04, 68.26, 53.04 and 65.22%, respectively. The enhanced OSC of RE–doped CeO_2_ should be attributed to the impurity–induced defects by the substitution of host Ce^4+^ with RE^3+^ into CeO_2,_ rather than the effects of its specific surface area and morphology.

## Figures and Tables

**Table 1:** Practical contents and nominal contents of RE in CeO_2_ synthesized using the hydrothermal process at 200 °C for 24 h and followed by calcination in air at 500 °C for 2 h (RE = Yb, Y, Sm and La).

RE in CeO_2_ (mol.%)	Yb			Y			Sm			La		
* Nominal contents	2	5	9	2	4	9	2	4	9	2	7	9
* Practical RE contents	2.58	5.26	9.62	1.92	4.24	8.67	2.41	4.27	9.38	2.19	6.79	9.25

* Nominal content (mol.%): $w\;(\mathrm{mol}.\%)=\frac{n_{RE}}{n_{RE}+n_{Ce}}\times 100,\;(n_{RE}+n_{Ce}=4_{}\;\mathrm{mmol})$. * Practical RE contents (mol.%): The actual RE doping amounts in CeO_2_ were determined using ICP–AES, where CeO_2_ was dissolved in a mixed solution of HNO_3_–H_2_O_2_.

**Table 2:** [Ce^3+^]_XPS_ and [V_O_]_XPS_ of Undoped CeO_2_ and 4 mol.% RE–doped CeO_2_ synthesized using solvothermal method at 200 °C for 24 h followed by calcination in air at 500 °C for 2 h (RE = Yb, Y, Sm and La).

	Sample	Undoped CeO_2_	4 mol.% RE–Doped CeO_2_			
Parameter			Yb	Y	Sm	La
[Ce^3+^]_XPS_ (%)		6.54	13.78	12.60	10.94	9.78
[*V*_O_]_XPS_ (%)		13.42	30.02	26.82	26.81	17.28
Specific surface area (m^2^/g)		96.0	89.7	98.1	112.6	104.6

## Data Availability

No new data were created.

## Sample Availability

Not available.
